# Subcutaneous Injection of Fowler’s Solution

**Published:** 1880-08

**Authors:** 


### SUBCUTANEOUS INJECTION OF FOWLER'S SOLUTION.

Dr. Moyes reports cases illustrative of special benefit derived from the *subcutaneous administration* of Fowler's solution. The first was a case of chronic pemphigus, which yielded but slowly to five minims of Fowler's solution three times a day, administered by the mouth. This was true of four attacks of the disease, occurring during four years, and each lasting about three months. A fifth attack was treated with hypodermic injections of Fowler's solution, and recovery took place in five weeks. In the former attacks the total amount of Fowler's solution taken averaged f ℥ij; in the last only f ʒ iijss were necessary to effect a cure. The method of administration was as follows: one-sixth grain of morphia was first injected in the usual way, and the needle was left stationary while the syringe itself was removed. Three to six minims of Fowler's solution, diluted with an equal quantity of water, were then drawn into the syringe, the latter was again attached to the needle, and the second injection was made. Immediately afterward a piece of ice was laid upon the part, and kept there for about half an hour by the patient himself, with the object of preventing inflammation. One injection was given daily. In this case no abscesses were produced. In a bad case of psoriasis, in which Fowler's solution had been for some weeks administered by the mouth, in considerable doses, with little benefit, improvement and cure soon followed hypodermic injections of five-minim doses, daily, of the same remedy. In this case some annoyance was caused by the development, at the point of injection, of two small abscesses. Professor McCall Anderson is reported to be making trial of the liquor sodii arseniatis, with the probability of its proving non-irritating, and not requiring the precaution of cold applications and previous morphine injection.

